# The heart of a dragon: 3D anatomical reconstruction of the ‘scaly-foot gastropod’ (Mollusca: Gastropoda: Neomphalina) reveals its extraordinary circulatory system

**DOI:** 10.1186/s12983-015-0105-1

**Published:** 2015-06-18

**Authors:** Chong Chen, Jonathan T. Copley, Katrin Linse, Alex D. Rogers, Julia D. Sigwart

**Affiliations:** Department of Zoology, University of Oxford, The Tinbergen Building, South Parks Road, Oxford, OX1 3PS UK; Ocean and Earth Science, University of Southampton, European Way, Southampton, SO14 3ZH UK; British Antarctic Survey, High Cross, Cambridge, CB3 0ET UK; Queen’s University Belfast, Marine Laboratory, Portaferry, BT22 1PF Northern Ireland

**Keywords:** ‘Scaly-foot gastropod’, *Chrysomallon squamiferum*, Morphology, Hydrothermal vents, Anatomy, Deep-sea, Adaptation, Neomphalina

## Abstract

**Introduction:**

The ‘scaly-foot gastropod’ (*Chrysomallon squamiferum* Chen et al., 2015) from deep-sea hydrothermal vent ecosystems of the Indian Ocean is an active mobile gastropod occurring in locally high densities, and it is distinctive for the dermal scales covering the exterior surface of its foot. These iron-sulfide coated sclerites, and its nutritional dependence on endosymbiotic bacteria, are both noted as adaptations to the extreme environment in the flow of hydrogen sulfide. We present evidence for other adaptations of the ‘scaly-foot gastropod’ to life in an extreme environment, investigated through dissection and 3D tomographic reconstruction of the internal anatomy.

**Results:**

Our anatomical investigations of juvenile and adult specimens reveal a large unganglionated nervous system, a simple and reduced digestive system, and that the animal is a simultaneous hermaphrodite. We show that *Chrysomallon squamiferum* relies on endosymbiotic bacteria throughout post-larval life. Of particular interest is the circulatory system: *Chrysomallon* has a very large ctenidium supported by extensive blood sinuses filled with haemocoel. The ctenidium provides oxygen for the host but the circulatory system is enlarged beyond the scope of other similar vent gastropods. At the posterior of the ctenidium is a remarkably large and well-developed heart. Based on the volume of the auricle and ventricle, the heart complex represents approximately 4 % of the body volume. This proportionally giant heart primarily sucks blood through the ctenidium and supplies the highly vascularised oesophageal gland. Thus we infer the elaborate cardiovascular system most likely evolved to oxygenate the endosymbionts in an oxygen poor environment and/or to supply hydrogen sulfide to the endosymbionts.

**Conclusions:**

This study exemplifies how understanding the autecology of an organism can be enhanced by detailed investigation of internal anatomy. This gastropod is a large and active species that is abundant in its hydrothermal vent field ecosystem. Yet all of its remarkable features—protective dermal sclerites, circulatory system, high fecundity—can be viewed as adaptations beneficial to its endosymbiont microbes. We interpret these results to show that, as a result of specialisation to resolve energetic needs in an extreme chemosynthetic environment, this dramatic dragon-like species has become a carrying vessel for its bacteria.

**Electronic supplementary material:**

The online version of this article (doi:10.1186/s12983-015-0105-1) contains supplementary material, which is available to authorized users.

## Introduction

Deep-sea hydrothermal vents represent a challenging environment where organisms face ‘extreme’ conditions such as hypoxia, very acidic water, and the presence of toxic materials such as hydrogen sulfide and heavy metals [1, 2]. Despite this, hydrothermal vents host a high biomass, comparable to that of tropical coral reefs (Van Dover, 2000). Vent ecosystems are supported by chemosynthetic primary production carried out by bacteria that oxidise reduced compounds such as hydrogen sulfide and methane to produce energy for fixing carbon dioxide or other carbon compounds into organic matter [3].

Animals flourishing in vents often have anatomical adaptations to the unusual environment. Many species host epibiotic microbes that they feed on for nutrition, for instance the vent shrimp *Rimicaris exoculata* Williams & Rona, 1986 houses both sulfur-oxidizing and methanotrophic bacteria in the gill chamber [4] and the galatheid squat lobster *Shinkaia crosnieri* Baba & Williams, 1998 farms bacteria on dense setae on the ventral face of the carapace [5]. Some species further house chemosynthetic bacteria internally as endosymbionts. The giant tube worm *Riftia pachyptila* Jones, 1981 is known to host thioautotrophic (sulfur-oxidizing) endosymbionts in a specialised organ, the trophosome; and has a vestigial digestive system [6]. Similarly, vesicomyid clams house endosymbionts in the gill and also have a much reduced digestive tract [7]. Some species are able to combine multiple feeding strategies, for example *Bathymodiolus thermophilus* Kenk & Wilson, 1985 is capable of filter-feeding while also hosting endosymbionts in the gill [8]. Other anatomical adaptations to thrive in chemosynthetic environments are also known. Many vent polychaetes have increased gill surface area to facilitate effective oxygen extraction, as well as high oxygen affinity haemoglobins and haemocyanins [9, 10].

The ‘scaly-foot gastropod’, *Chrysomallon squamiferum* Chen et al., 2015 [11], is distinctive among hydrothermal-vent molluscs for its numerous dermal sclerites, which are often mineralised with iron sulfide. This species inhabits the hydrothermal vent fields of the Indian Ocean, on the walls of black-smoker chimneys or directly on diffuse flow sites. First discovered in the Kairei hydrothermal vent field, Central Indian Ridge (CIR) [12], it has also been found in two more vent fields: Solitaire field, CIR (Nakamura *et al*., 2012) and Longqi field (literally meaning ‘dragon flag’ in Chinese, and also known as Dragon field, [13, 14]), Southwest Indian Ridge (SWIR) [13, 15, 16]. Both morphological and genetic characters place *C. squamiferum* in Peltospiridae, a family restricted to hydrothermal vent ecosystems in the clade Neomphalina [17]. *Chrysomallon squamiferum* reaches 45 mm in shell length, much larger than the other members of the family which are generally small (<15 mm in shell length).

The unusual external morphology of *Chrysomallon* has been described by a number of studies [17–19] with the surface mineralogy investigated in detail [20, 21], and a number of studies have concentrated on its unique way of housing endosymbionts in the oesophageal gland [22, 23], instead of the gill, like other vent gastropods such as *Alviniconcha* spp. [24]. The internal anatomy of several peltospirid genera have been investigated previously (mainly *Rhynchopelta concentrica* McLean, 1989 but also *Peltospira*, *Nodopelta*, *Echinopelta* and *Hirtopelta* [25]; as well as *Pachydermia laevis* Warén & Bouchet, 1989 [26]), and *C. squamiferum* is known to differ from these, particularly in its acquisition of endosymbionts and gigantism relative to other peltospirids [17, 22]. The only previous description of the internal anatomy of the ‘scaly-foot gastropod’ is a short supplementary text [17]. The aim of the present study was to investigate this unusual species to examine possible further anatomical adaptations to life in the vent environment.

## Results

**Fig. 1:**
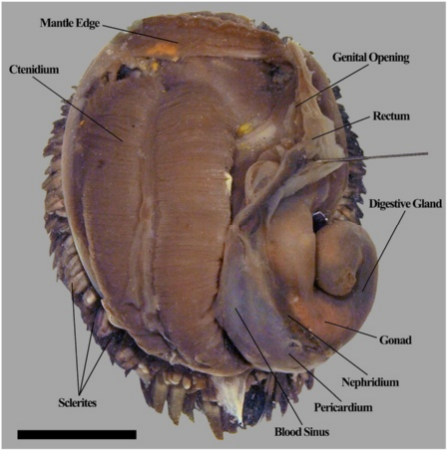
The ‘scaly-foot gastropod’, *Chrysomallon squamiferum*, mantle cavity overview (shell and mantle tissue removed). Scale bar: 1 cm

**Fig. 2:**
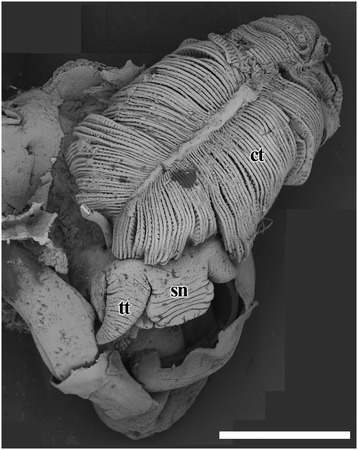
Structural details of the head and ctenidium of *Chrysomallon squamiferum*, composite of 22 scanning electron micrographs of a freeze-dried juvenile specimen. Abbreviations: ct, ctenidium; sn, snout; tt, cephalic tentacle. Scale bar: 2 mm

**Fig. 3:**
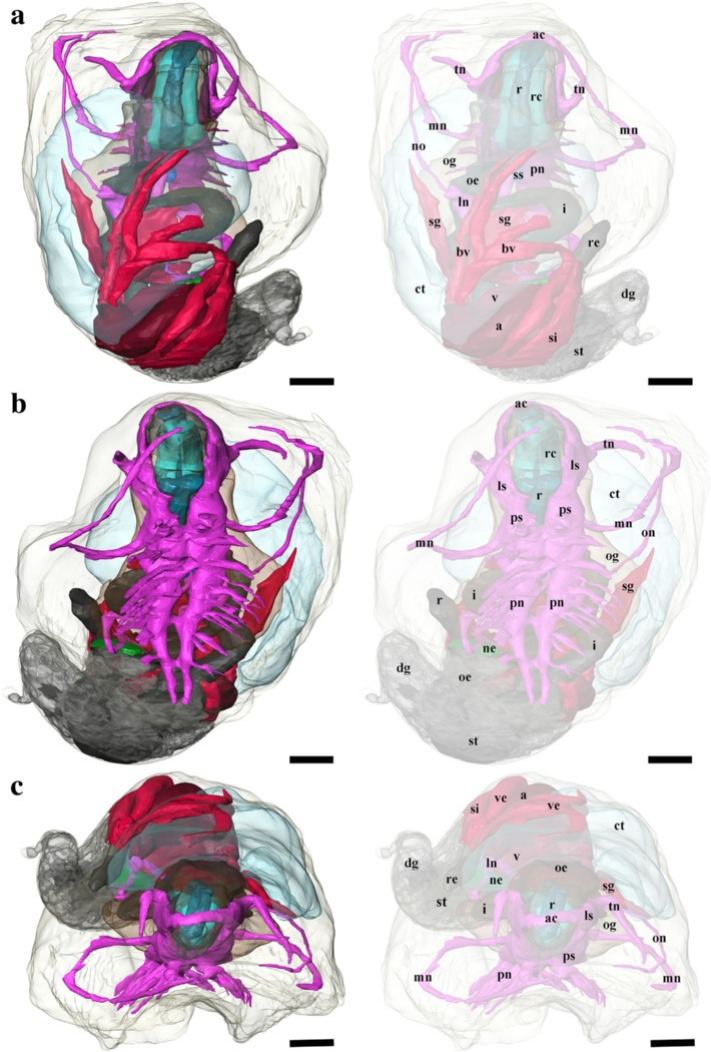
3D tomographic reconstruction of *Chrysomallon squamiferum*, the full anatomical model in various views. Soft body outline (mantle and foot) shown in transparency. Ctenidium, anterior oesophagus, oesophageal gland, and digestive gland are rendered semi-transparent to show structures underneath. For all parts, the tomographic model is shown to left and a second copy of the same view with labelled parts shown to right. **a**. Dorsal view. **b**. Ventral view. **c**. Frontal view. Each colour group corresponds to a specific anatomical system: grey/black, digestive tract; green, oesophageal gland; translucent blue, ctenidium; red, circulatory system (excluding ctenidium); fuchsia, nervous system. Abbreviations: a, auricle; ac, anterior commissure; ct, ctenidium; dg, digestive gland; i, intestine; ln, lateral (visceral) nerve; ls, lateral swelling; mn, mantle nerve; ne, nephridium; oe, oesophagus; og, oesophageal gland; on, osphradial nerve; pn, pedal nerve; ps, pleural swelling; r, radula; rc, radula cartilage; re, rectum; sd, duct connecting stomach to digestive gland; sg, blood sinus under the ctenidium; si, blood sinus; ss, statocyst; st, stomach; tn, tentacular nerves; v, ventricle; ve, blood vessel. Scale bars: 250 μm

**Fig. 4:**
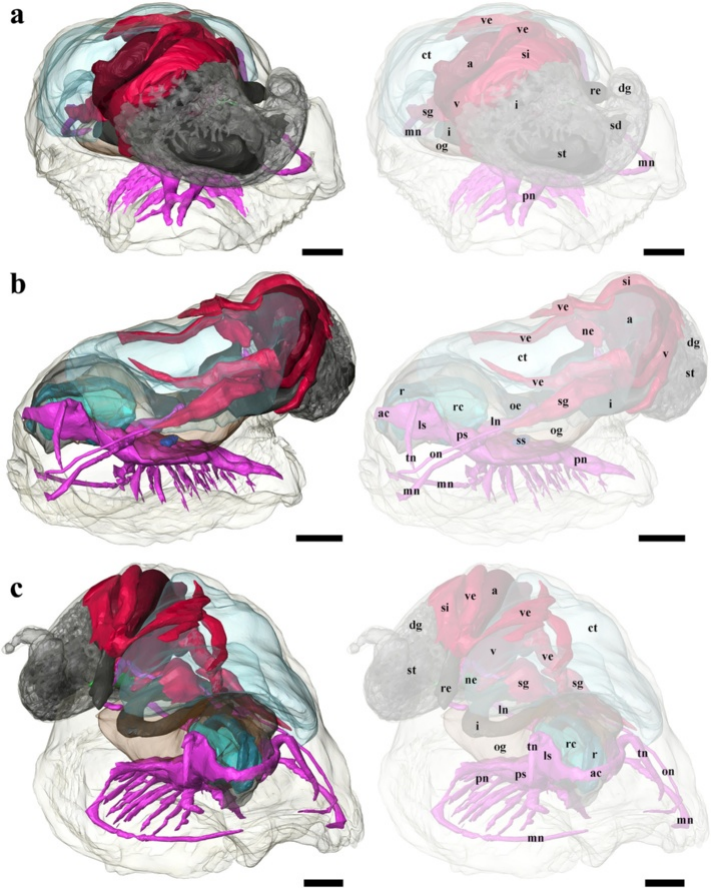
3D tomographic reconstruction of *Chrysomallon squamiferum*, the full anatomical model in various views (continued). Soft body outline (mantle and foot) shown in transparency. Ctenidium, anterior oesophagus, oesophageal gland, and digestive gland are rendered semi-transparent to show structures underneath. For all parts, the tomographic model is shown to left and a second copy of the same view with labelled parts shown to right. **a**. Rear view. **b**. Left view. **c**. Angled (right side) view. Each colour group corresponds to a specific anatomical system: grey/black, digestive tract; green, oesophageal gland; translucent blue, ctenidium; red, circulatory system (excluding ctenidium); fuchsia, nervous system. Abbreviations: a, auricle; ac, anterior commissure; ct, ctenidium; dg, digestive gland; i, intestine; ln, lateral (visceral) nerve; ls, lateral swelling; mn, mantle nerve; ne, nephridium; oe, oesophagus; og, oesophageal gland; on, osphradial nerve; pn, pedal nerve; ps, pleural swelling; r, radula; rc, radula cartilage; re, rectum; sd, duct connecting stomach to digestive gland; sg, blood sinus under the ctenidium; si, blood sinus; ss, statocyst; st, stomach; tn, tentacular nerves; v, ventricle; ve, blood vessel. Scale bars: 250 μm

**Fig. 5:**
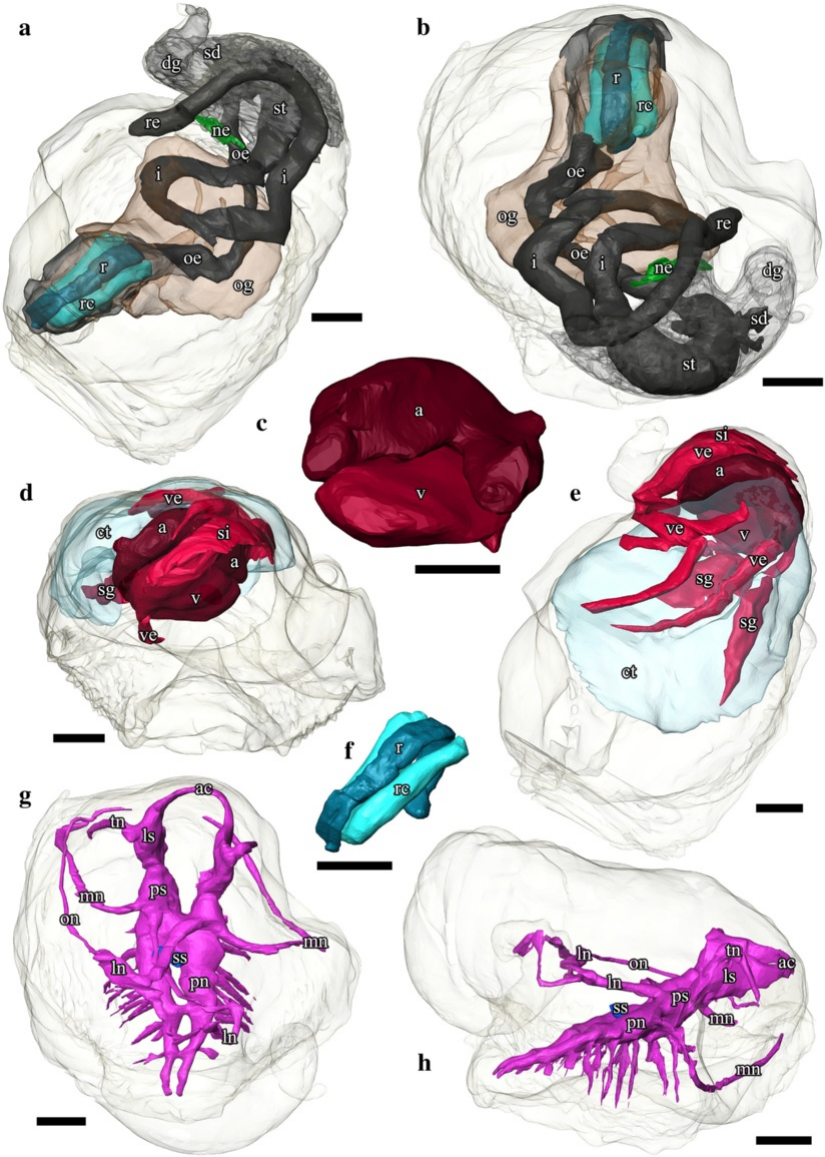
3D tomographic reconstruction of *Chrysomallon squamiferum*. Soft body outline shown in transparency. Ctenidium, anterior oesophagus, oesophageal gland, and digestive gland are rendered semi-transparent to show structures underneath. **a**-**b**. Digestive and excretory systems. **c**. Heart. **d**-**e**. Circulatory system. **f**. Radula and radula cartilage. **g**-**h**. Nervous system. Abbreviations: a, auricle; ac, anterior commissure; ct, ctenidium; dg, digestive gland; i, intestine; ln, lateral (visceral) nerve; ls, lateral swelling; mn, mantle nerve; ne, nephridium; oe, oesophagus; og, oesophageal gland; on, osphradial nerve; pn, pedal nerve; ps, pleural swelling; r, radula; rc, radula cartilage; re, rectum; sd, duct connecting stomach to digestive gland; sg, blood sinus under the ctenidium; si, blood sinus; ss, statocyst; st, stomach; tn, tentacular nerves; v, ventricle; ve, blood vessel. Scale bars: 250 μm

**Fig. 6:**
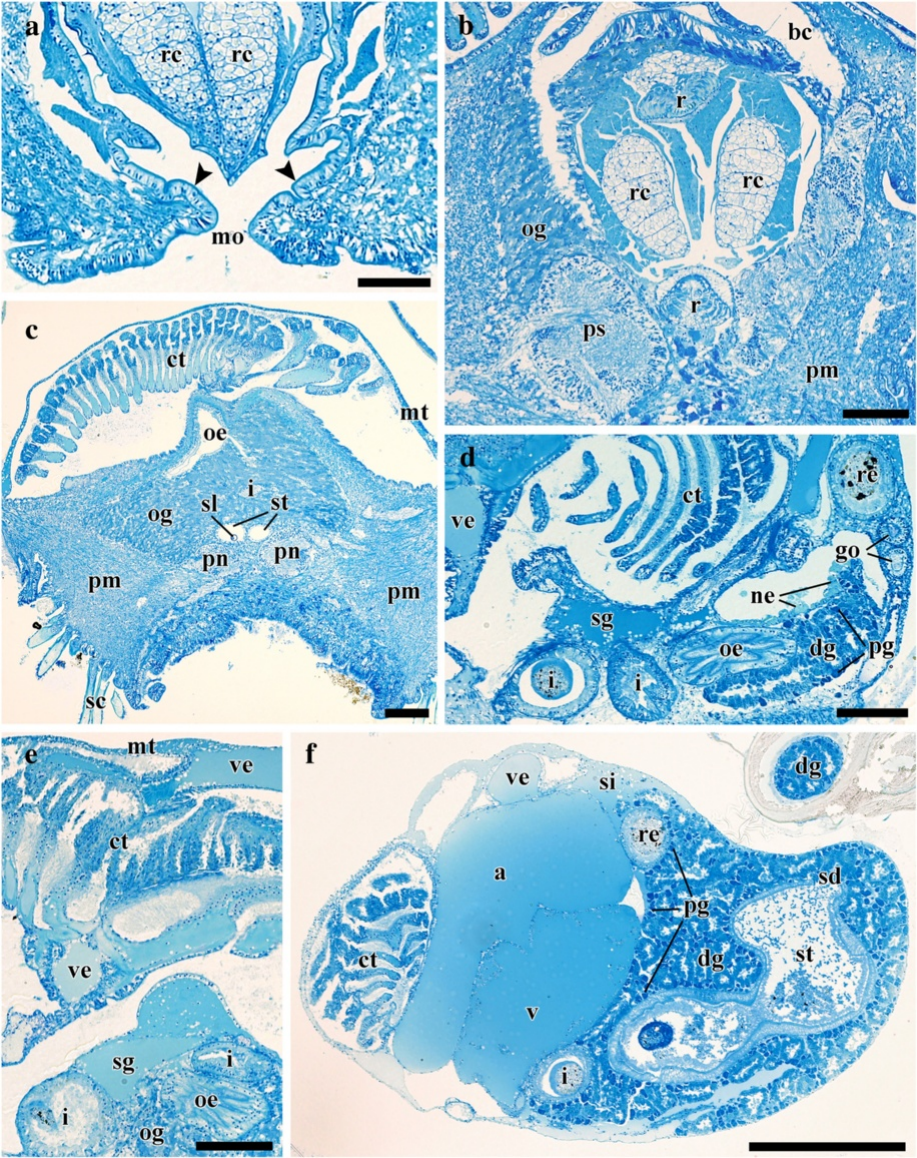
Transverse semi-thin sections from *Chrysomallon squamiferum*. **a**. Mouth and buccal mass showing the epithelium cuticle (black arrows). **b**. Posterior part of the head showing the large fused neural mass. **c**. Mid-body section showing ctenidium, oesophageal gland, pedal nerves, and statocysts. **d**. Anterior of the heart. **e**. Ctenidium and associated blood vessels and sinuses. **f**. Posterior visceral mass. Abbreviations: a, auricle; bc, buccal cavity; ct, ctenidium; dg, digestive gland; go, gonoduct; i, intestine; mo, mouth; mt, mantle; ne, nephridium; oe, oesophagus; og, oesophageal gland; pg, putative proto-gonad; pm, pedal musculature; pn, pedal nerve; ps, pleural swelling; r, radula; rc, radula cartilage; re, rectum; sc, sclerites; sd, duct connecting stomach to digestive gland; sg, blood sinus under the ctenidium; si, blood sinus; sl, statolith; ss, statocyst;st, stomach; v, ventricle; ve, blood vessel. Scale bars: 100 μm

**Fig. 7:**
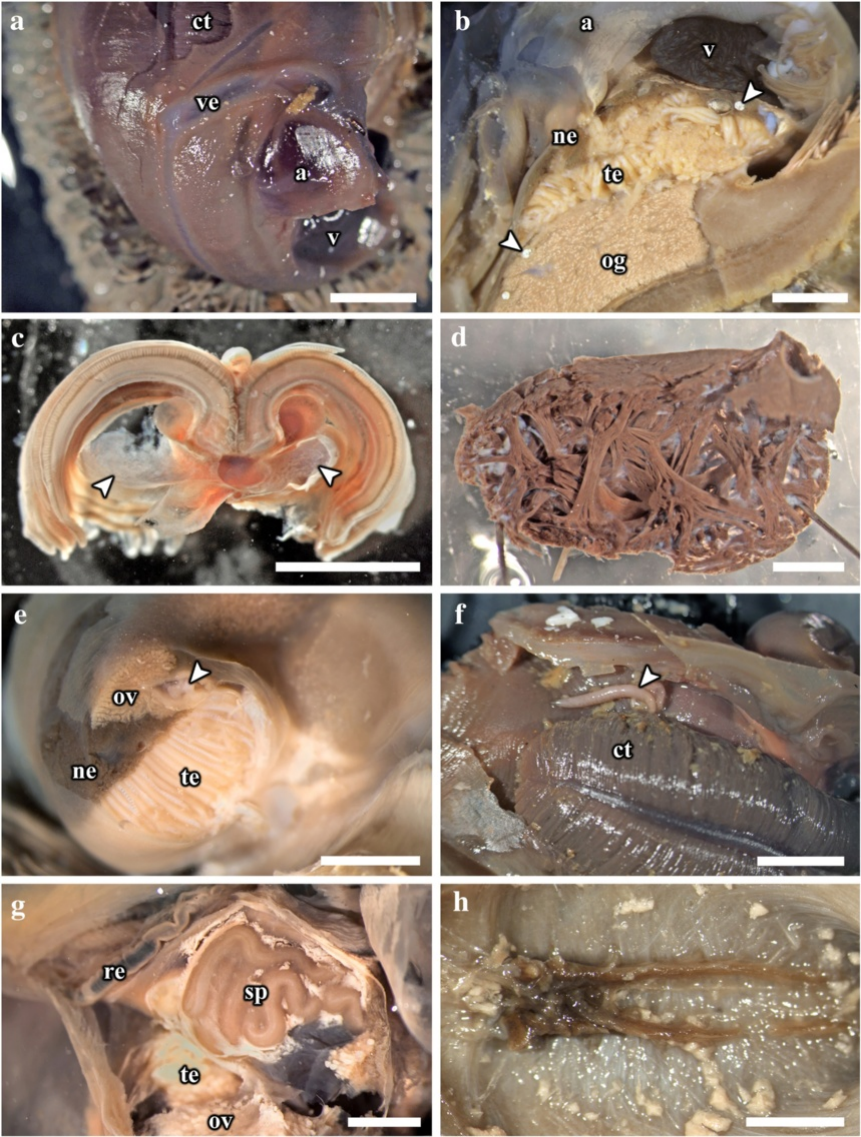
Photographs of the soft parts of adult *Chrysomallon squamiferum*. **a**. Dorsal view with shell and part of mantle removed, showing the ctenidium, heart, and associated blood vessels. **b**. Sagittal section; white arrows indicate sections of the hindgut showing white, chalky material inside. **c**. Transverse section through the ctenidium showing a pair of blood sinus (white arrows). **d**. Sagittal section through ventricle showing muscle bundles. **e**. Transverse section of the visceral mass showing testis, ovary, and nephridium; white arrowhead indicates spermatophores inside the ‘spermatophore producing organ’. f. Packaged spermatophore (white arrow). g. ‘Spermatophore producing organ’. h. Pedal nerve cords, fused neural mass at anterior and numerous lateral offshoots distally into the foot. Abbreviations: a, auricle; ct, ctenidium; ne, nephridium; og, oesophageal gland; ov, ovary; re, rectum; sp, ‘spermatophore producing organ’; te, testis; v, ventricle; ve, blood vessel. Scale bars: a-c, e-f, h = 5 mm, d, g = 2 mm

The overall anatomical plan of *Chrysomallon squamiferum* conforms to that of other Neomphalina (Figs. 1, 2). This include non-papillate tentacles, single left bipectinate ctenidium, single auricle, rhiphidoglossate radula with a single pair of radular cartilages, a rectum that does not penetrate the heart but passes ventral to it, and statocyst with a single statolith (Figs. 3, 4, 5; [27]). A number of histological characters can also be compared to other members of Neomphalina (Fig. 6). The basic organ anatomy in adult specimens was previously described by Warén *et al.* [17] but our dissections expand considerably on that report (Fig. 7).

### External Morphology

*Chrysomallon squamiferum* is a loosely coiled snail with the soft parts occupying approximately two whorls (Figs. 1, 3a). The snout is thick and tapers distally to a blunt end, the mouth is a circular ring of muscles when contracted and closed. Two smooth cephalic tentacles are present, these are thick at base and gradually tapers to a fine point at the distal tips (Fig. 2). There are no eyes. Specialised copulatory appendages are lacking in the anterior head-foot region. There is no pedal gland in the propodium. The epipodium does not carry any epipodial tentacles but is instead densely covered in hard sclerites, imbricating in a roof-tile manner. An operculum is present at the metapodium, exposed and well-sized in juveniles (Additional file 1: Fig. 1) but the relative size decreases as individuals grow. In adults the operculum is elongate and clearly distinct but buried under layers of sclerites ([19]: fig. 2). The shell muscle is horse-shoe shaped and large, divided in two parts on the left and right, connected by a narrower attachment. The mantle edge is thick but simple without distinct features. The mantle cavity of *C. squamiferum* is deep and reaches the posterior edge of the shell. The medial to left side of the cavity is dominated by a very large bipectinate ctenidium (Figs. 1, 2, 3a). Ventral to the visceral mass the body cavity is occupied by a huge oesophageal gland extended to fill the ventral floor of the mantle cavity (Fig. 4c).

### Digestive and Excretory System

The mouth opening on the ventral side of snout leads to buccal cavity contains a rhipidoglossate radula (Fig. 5a). The epithelium of the inner lip is composed of a columnar epithelium with a thin cuticle (Fig. 6a). There are no jaws. We found no discrete salivary glands. The radula ribbon is long, width to length ratio is approximately 1:10 in the serially sectioned specimen (Figs. 3c, 5b; structural details shown by Warén *et al.* [17]: fig. S2A). A single pair of prominent radula cartilages support the anterior radula ribbon. The two cartilages are in contact at their anterior extent and become separate ventrally (Fig. 5f). At the posterior end of the cartilage pair the radula ribbon folds under and emerges from the growing radular sac below the buccal mass. A part of the anterior oesophagus rapidly expands into a huge hypertrophied blind-ended oesophageal gland (Fig. 5a) which occupies much of the ventral face of the mantle cavity (estimated 9.3 % body volume). The dorsal fold of the oesophagus (Fig. 6b) may represent an extensive food groove but we cannot exclude the possibility this shape is due to an artefact of tissue distortion during fixation. The oesophageal gland has a uniform texture (Fig. 6c) and is highly vascularised with fine blood vessels (Fig. 7b).

The digestive system apart from the oesophageal gland is relatively small and forms a simple loop (Fig. 5b). The midgut is forms a clearly discernable stomach as a distinctly widened and enlarged region separating it from the foregut and hindgut (Fig. 5b). There is no gastric shield nor style; the stomach wall is ciliated but we could not identify a discrete sorting area. The stomach has at least three ducts at its anterior right connecting to the digestive gland; the extensive and unconsolidated digestive gland extends to the posterior filling the shell apex (Fig. 2d, 2f). The nephridium is central, tending to the right side of the body (Figs. 1, 5b), as a thin dark layer of glandular tissue (Fig. 7e). It is anterior and ventral of the digestive gland and in contact with the dorsal aspect of the foregut (Fig. 5a).

The posterior aspect of the oesophagus passes directly above the oesophageal gland and ventral of the auricle to reach the ventral face of the visceral mass (Fig. 5a). The digestive system runs directly from anterior to posterior in contact with the dorsal aspect of the oesophageal gland, and the nephridium (immediately posterior to the oesophageal gland); it widens into a distinct stomach embedded within the digestive gland. The posterior intestine emerges from the left anterior side of the stomach, runs anteriorly and curving to the right into the oesophageal gland and the next turn of the digestive tract is completely embedded within the oesophageal gland. It then returns out of the oesophageal gland to the left, crossing dorsally over the oesophagus, runs posteriorly to contact the digestive gland dorsal of the stomach, and then turns anteriorly down the right mantle wall to exit the mantle cavity on the right, as the rectum (Figs. 3a, 7g). The anus opening is also located on right side of animal, close to the transverse mid-line of the gastropod, above the genital slit (Fig. 1). The intestine throughout is simple in cross section and does not have a typhosole.

The hindgut was typically filled with consolidated pellets of chalky unidentified material, only present in stomach and posterior to it, and seen in specimens of all sizes (Figs. 6f, 7b). These are possibly sulphur granules produced by the endosymbiont and represent a way for detoxing hydrogen sulfide.

### Circulatory System

The single large ctenidium (Figs. 1, 5e) occupying 15.5 % of the body volume in the serially sectioned specimen), it reaches the posterior end of the mantle cavity and further curls to enter the visceral mass. It is densely packed with thin gill filaments on both sides (Figs. 2, 6c) and apparently without skeletal rods. There are two prominent semi-enclosed blood sinuses under the gill (0.7 % body volume), which fill the concavity formed by the ventral aspect of the gill filaments (Figs. 5e, 6e, 7c). To the posterior right of the ctenidium lies a hypertrophied auricle (Figs. 5c, 6f). A ventricle of even larger size lies directly ventral of the auricle (Fig. 7a). A simple pericardium is present (not illustrated) and encloses the heart with tissue connected to the dorsal mantle. The ventricle has thick muscular walls with many crossing muscle bundles (Fig. 7d). The auricle and ventricle together occupy 4 % of the body volume (Figs. 5d, 6f). Diagonally above the right half of the auricle is a prominent blood sinus directly connected to the auricle (0.8 % body volume), it begins anteriorly before the anterior end of the auricle and extends posteriorly to reach posterior end of the ventricle (Fig. 5c). Thick blood vessels are clear above and below the ctenidium (Fig. 5e). In adult specimens several blood sinuses are located in the posterior half of the mantle cavity but the location and extent differed between individuals. Some of these blood sinuses are connected to the mantle tissue but other large areas of haemocoel, jellylike and with a pale blue-grey colour in preserved specimens, are found covering the gonad and throughout the body (Fig. 1). The oesophageal gland is highly vascularised with smaller blood sinuses and a dense network of fine blood vessels (Fig. 7b).

### Nervous System and Sensory Structures

The nervous system is voluminous (Figs. 5g, 5h; occupying approximately 5.7 % of body volume in the serially sectioned juvenile specimen, although proportionately much smaller in adults). The brain is a more or less solid neural mass without clear structure or ganglia, and there are no eyes or other cephalic sensory structures. As typical of gastropods the nervous system is composed of an anterior oesophageal nerve ring and two pairs of longitudinal nerve cords, with the ventral pair innervating the foot and the dorsal pair forming a twist via streptoneury. The oesophageal nerve ring and pedal nerves are thickened and medullary in nature (Fig. 6b). There is no clear structural evidence from juvenile or adult specimen for the identification of specific ganglia (*sensu* Richter *et al.* [28], *i.e.* nerve tissue in discrete units separated by an area containing no cell bodies), although the anterior region of the nervous system shows some intergressions of cell bodies into the central neuropil that appear to divide the mass into regions or lobes, the neural masses appear to be wholly fused and without ganglionic structure.

The anterior commissure, or frontal aspect of the oesophageal nerve ring, is large, connecting two lateral swellings (Fig. 5h). Laterally from each of these swellings is a large, prominent tentacular nerve projecting into the cephalic tentacles. There are no eyes and no evidence of even a remnant optic nerve emerging from the oesophageal nerve ring. At the posterior margin of the oesophageal nerve ring is a pair of prominent pleural swellings. From each pleural swelling a long nerve goes anteriorly along the margin of the mantle around the head.

Both the lateral (visceral) and pedal nerves, as well as a pair of mantle nerves, emerge from the posterior aspect of the oesophageal nerve ring more or less co-located in a large junction (Figs. 5f, 6b). The pedal nerves are fused in a large body and extend into two thick medullary cords, separating posteriorly into two clear cords (Fig. 6c) but joined at several points by bridging commissures. The pedal nerves are not embedded within the foot musculature but sit on the dorsal aspect of the foot muscle block, below the oesophageal gland mass (Fig. 7h). A number of branching nerves penetrate distally between muscle fibres into the muscle block of the foot, and the main pair of pedal nerves penetrate the ventral wall of the body cavity into the foot at the posterior (Figure 3b). A pair of statocysts are located medially, anterior of the pedal nerve cords, apparently innervated by them, more or less central in the animal body (Fig. 5h). The statocysts are surrounded by the oesophageal gland; each statocyst contains a single statolith (Fig. 6c).

The left lateral nerve cord is embedded within the oesophageal gland, passing underneath the intestinal loop and emerging at the right posterior, continuing to the nephridial tissue (Fig. 4c). The right lateral nerve cord crosses through the oesophageal gland above the left lateral nerve and emerges between the foregut and the hindgut underneath the gill. At this point a small nerve, positionally similar to the osphradial nerve in some other gastropods, splits from the main lateral nerve cord and runs anteriorly to the anterior mantle (Figs. 4b, 5f). The right lateral nerve posterior of this point continues above the digestive tract and below the gill and runs posteriorly to meet the terminus of the left lateral nerve cord.

A small pigmented patch is present at the right side of the gill stem at the anterior of the ctenidium; we tentatively compare this to the osphradium of other gastropods. This area could not been identified in the tomographic reconstruction of the juvenile specimen, and the putative osphradial nerve could not be seen in adult specimens. We confirm the ctenidial bursicles observed by Warén et al. ([17]: fig. S2C) but could not identify these histologically from the juvenile specimen.

### Reproductive System

*Chrysomallon squamiferum* is a simultaneous hermaphrodite. Adults possess both testis and ovary, although the level of development of the two varied in different individuals. The gonads are arranged as two discrete layers with the nephridial tissue between them, the testis to the ventral and the ovary dorsal (Fig. 7e).

A large organ is present on the distal body cavity on the right side of the animal as a distinct complex twisted duct fused into a disc distal of the testis, and this is associated with the male gonoduct, (Fig. 7g). We were not able to confirm spermatophores being packaged inside this organ (Fig. 7e). A packaged spermatophore was confirmed to be discharged from the gonopore of one individual (Fig. 7f), providing further evidence for internal fertilisation through transfer of spermatophore packets. Gonoducts from the testis and ovary are initially separate but apparently fuse to a single duct and emerge as a single genital opening on the right of the mantle cavity, ventral and anterior of the rectum (Fig. 1). This was also seen in the serially sectioned juvenile specimen (Fig. 6d), although this could not be traced as there was no mature gonad present, only putative proto-gonad found associated with the digestive gland (Fig. 6f). The genital opening in adults is simple, not associated with any specialised copulatory organs or appendages, and there are no penis modified from cephalic tentacles. In adults the gonads are displaced out of the shell apex into to the head-foot region at the right side (Fig. 1).

## Discussion

Members of the gastropod clade Neomphalina are endemic to reducing environments in the deep sea, primarily living on hydrothermal vents (all Peltospiridae) but with some species on natural deposits of sunken wood [29]. The comprehensive anatomical framework presented in the present study allows us to consider both evidence for the phylogenetic affinity of *Chrysomallon* and also the nature of its particular anatomical characters.

Given the uniqueness of *Chrysomallon's* anatomy we suspect that this highly autapomorphic condition is the result of numerous adaptations associated with life in the acidic flow of hydrothermal vents. The anatomy of several other genera in Neomphalina has been described in detail through either dissection (e.g., [25]) or tomographic reconstruction [27]. The diversity of taxa with well-characterised anatomy encompasses *Chrysomallon* and members of all three families currently recognised in Neomphalina: in Peltospiridae mainly *Rhynchopelta*, *Peltospira* ([25]; also includes brief overview of *Nodopelta, Echinopelta*, and *Hirtopelta*), as well as *Pachydermia* [26]; in Neomphalidae *Neomphalus* [30] and *Symmetromphalus* [31]; in Melanodrymiidae *Melanodrymia* [32], *Leptogyra*, and *Leptogyropsis* ([27], with mentions of *Xyleptogyra*). The present study is the first to include both of these approaches to encompass comprehensive description across full post-settlement ontogeny.

The gross anatomy of *Chrysomallon* generally conforms to the neomphaline plan [27]. Warén *et al*. ([17]: fig. S2C) showed sensory bursicles on the tip of the gill filaments which are known to be present in most vetigastropods and present some neomphalines [27, 32], though the majority of taxa lack them (e.g., *Pachyermia laevis* [26]; *Lirapex* [33]). The lack of specialised copulatory organs in *Chrysomallon* conforms to Peltospiridae; members of the family Neomphalidae have the left cephalic tentacle modified as a penis [25, 31, 34] and melanodrymiids have various special cephalic copulatory modifications [27, 32]. However, as a member of Peltospiridae, *Chrysomallon* is the only taxon in that family so far known to be a simultaneous hermaphrodite.

### Comparative anatomy

The circulatory system of *Chrysomallon* is notably enlarged compared to other gastropods, as was briefly mentioned by Warén *et al*. [17]. On dissection, the blood sinuses and lumps of haemocoel material are a prominent feature throughout the body cavity. A cephalopedal haemocoel lined by a net of lacunae reported by Warén *et al*. [17] could not be found, but the blood sinuses are large and apparently mobile as they differ in position among individuals. The bipectinate ctenidium extends far behind the heart into the upper shell whorls, which is much larger compared to *Peltospira* with a similar shell shape and general form as well as other peltospirids [25, 26] or melanodrymiids [27, 32]. It is similar, however, in proportional size to *Hirtopelta* which has the largest gill among peltospirid genera investigated anatomically so far [25, 35]. An enlarged gill may be associated with filter-feeding (as is shown for neomphalid genera *Neomphalus* [30] and *Symmetromphalus* [31]), symbiotic bacteria on the gill (such as endosymbionts in *Hirtopelta tufari* Beck, 2002 [35] or *Alviniconcha* spp. provannids [36]), and/or high respiratory demand. There are no endosymbionts in the gill of *Chrysomallon* [22].

Given that *Chrysomallon squamiferum* hosts endosymbionts in the oesophageal gland, has no symbionts in or on the gill [22], and probably does not filter-feed, the most likely reason for enlargement of the gill is to fulfill raised respiratory needs. Nakagawa *et al.* [23] showed through whole-genome sequencing that the endosymbionts of C. *squamiferum* are thioautotrophic gammaproteobacteria with a full set of genes required for aerobic respiration, and probably capable of switching between more efficient aerobic respiration and less efficient anaerobic respiration depending on oxygen availability. The host also requires oxygen for survival, and the enlargement of gill is also likely to facilitate extracting oxygen from low oxygen conditions typical of hydrothermal vent ecosystems [1]. Being a coiled snail *Chrysomallon* is probably unable to increase respiratory surface area by increasing the mantle surface, as seen in many limpet form gastropods [37].

The most exceptional part of the circulatory system is the extremely large monotocardian heart, which has an especially well-developed ventricle with very thick muscular walls reinforced by muscle bundles running across the lumen. A ventricle with thick muscular wall is known in Peltospiridae from *Rynchopelta concentrica* [25], but the proportional size of heart is greater in *Chrysomallon*. In neomphalids the heart is not markedly muscular (*Neomphalus*, [30]). Unlike *Pachydermia laevis* where the auricle is larger than the ventricle [26], the ventricle is even larger than the auricle in *Chrysomallon squamiferum*. Large blood vessels dorsal and ventral to the ctenidium, together with numerous large blood sinuses under the gill and in the mantle cavity and the fact that the oesophageal gland is highly vascularised, indicate that the giant heart primarily serves the ctenidium and the oesophageal gland (also briefly noted by Warén *et al*. [17]).

The heart is unusually large for a peltospirid (compare to [25]) or indeed any animal proportionally. The heart of *Chrysomallon* is estimated at 4 % of the body volume; by contrast the heart of a healthy human is averages at around 1.3 % of the body volume (mean total heart volume of adult humans 778 ml, taken from [38]; mean human body volume 61.05 L, average of both genders [39]). We interpret the heart, and particularly the muscular ventricle, functions to create suction that draws blood through the gill and thus pump haeomocoel to the rest of the circulatory system.

An oesophageal gland or pouch is a common feature in so-called basal gastropod clades, including Patelloidea, Vetigastropoda, Cocculiniformia, Neritimorpha and Neomphalina [40], but the extent of enlargement seen in *Chrysomallon* is orders of magnitude greater than any other gastropod anatomy described to date [17]. In other peltospirids, the posterior portion of the oesophagus forms a pair of blind mid-oesophageal pouches or gutters extending only to the anterior end of the foot (*Rynchopelta*, *Peltospira*, *Nodopelta*, *Echinopelta*, [25]: fig. 12; *Pachydermia* [26]; also *Melanodrymia* [32]). The oesophageal gland of *Chrysomallon* forms one single voluminous blind sac that extends much further posterior, to reach the heart. The oesophageal gland is known definitively to house one single strain of thioautotrophic endosymbiotic bacteria [22, 23]. There are no other reports of oesophageal glands that are highly vascularized and containing blood sinuses as seen in *Chrysomallon*. Two families of limpet formed gastropods, Bathyphytophilidae and Lepetellidae, with members inhabiting chemosynthetic environments are also known to have enlarged oesophageal pouches, however, though not to the extent of *Chrysomallon* [41, 42]. Both are known to house endosymbiotic bacteria, in the case of bathyphytophilids most likely also in the oesophageal glands [41, 42] but in the lepetellids the endosymbionts are spread in the haemocoel [42]. The dominance of the greatly enlarged oesophageal gland housing endosymbiotic bacteria is in contrast with rest of the digestive system which is relatively small, suggesting that the endosymbionts are the key nutrient source. We further infer the function of the enlarged circulatory system and extremely high blood volume is relevant to the metabolism of the bacterial endosymbionts.

Most holobiont vent molluscs such as *Calyptogena*, *Alviniconcha* and *Ifremeria* house endosymbionts in the gill where bacteria can readily contact vent fluid through circulation in the mantle cavity [1, 36, 43]. In *Calyptogena* clams, for example, the bacteriocytes containing the endosymbionts are situated on the surface of gill filaments and have an absorptive end exposed to the mantle cavity. Vent fluid containing hydrogen sulfide is circulated in the mantle cavity and the endosymbionts simply take up the sulfide from this fluid [1, 44, 45]. In *Chrysomallon*, however, the endosymbionts are housed in oesophageal gland where they are isolated from the vent fluid. The host is thus likely to play a major role in supplying the endosymbionts with necessary chemicals leading to increased respiratory needs, similar to the scenario in the trophosome of *Riftia pachyptila*. To supply endosymbionts with sulfides, *R. pachyptila* takes in sulfides from the vent fluid through its plume and has hydrogen sulfide specific binding site on the haemoglobin molecule in the blood that transports sulfides to the trophosome [46]; such sulfide-specific binding site is lacking in the haemoglobin of *Calyptogena* [1, 44, 45]. The same may be happening with *Chrysomallon* and an elaborative cardiovascular system with a powerful heart is likely to help circulate sufficient oxygen or hydrogen sulfide through the blood stream, for the needs of the host (oxygen) and its symbiotic bacteria (oxygen and sulfide). Detailed investigation of the haemocoel of *C. squamiferum* will reveal further information regarding its oxygen carriers and if it has respiratory pigments that bind to sulfides or an alternative means of sulfide transport.

The stomach of *Chrysomallon* is similar in form to other neomphalines including the tubes we observed in the juvenile specimen that connect the stomach directly to the digestive gland ([30]: fig. 6). The intestine however is reduced [17] and only has a single loop, unlike grazing peltospirids [25, 26], most likely because of its nutritional reliance on endosymbiotic bacteria. *Hirtopelta* also has a reduced and narrow intestine [25], but members of that genus house endosymbionts on their enlarged gill filaments [35], and the intestinal reduction is likely to also be a result of convergence due to reliance on endosymbionts for nutrition. In filter-feeding neomphalids the intestine is also rather short (*Neomphalus* and *Symmetromphalus*); the gills of these are enlarged as well, but it is unknown if they house symbionts on the gill filaments. Melanodrimiids are detritivores and have longer intestines like grazing peltospirids [27, 32]. There is a general trend associating short simple guts with reliance on endosymbiosis rather than external nutrition through grazing.

Gut contents from *C. squamiferum* specimens did not include any coarse inorganic particles such as those commonly found ingested together with food by grazing or deposit feeding peltospirids such as *Rhynchopelta concentrica* [25]. Warén *et al*. [17] reported finely granular sulfides from the gut of *C. squamiferum* and suggested that these likely originated from the endosymbionts; we also confirmed these sulfides. The material nature of the predominant chalky material in the posterior region of the gut is still unclear. The chalky gut content may represent ingested endosymbionts or other food source (such as filter-feeding), it is currently unclear whether this species has other mechanisms of feeding.

The radula of *C. squamiferum* is proportionately much larger in juveniles compared with adults. The animals could feed by grazing as juveniles; there is also a putative shift in diet reported from *Neomphalus* where juveniles feed by grazing and shift to exclusive filter-feeding in later life [30]. However the material in the gut of the serially-sectioned juvenile *C. squamiferum* contained similar material to the adult guts, the foregut of the juvenile was also empty and the oesophageal gland was proportionately as large in the juvenile as in adults. This suggests *C. squamiferum* likely relies on endosymbionts for nutrition throughout its entire post-larval life. The endosymbionts are remarkably similar in their genome with only two synonymous changes in 19 genes and 13810 codon positions, across 32 host individuals [23]. Nakagawa *et al*. [23] suggested this is a result of stringent selection of horizontally transferred endosymbiont by the host.

No other neomphalines are known to have endosymbionts housed in the oesophageal gland, although *Hirtopelta tufari* is known to house endosymbionts in the ctenidium (Beck, 2002) in a similar association as found in provannid gastropod species in *Alviniconcha* and *Ifremeria* [29]. Another undescribed peltospirid from Antarctic vents at East Scotia Ridge of similar body size to *C. squamiferum* (‘Peltospiroid n. sp. ESR’, [47]) has been shown through stable isotope analyses to possibly be reliant on endosymbionts for nutrition [48].

The single nephridium in *C. squamiferum* is displaced to the right of the body, similar to the structure seen in *Hirtopelta* ([25]: fig. 15). This may be attributable to the enlarged gill taking up the entire left side of mantle cavity, seen in both taxa. The left side of the body in *C. squamiferum* has numerous voluminous blood sinuses that occupy all available space.

The arrangement of the nervous system in *C. squamiferum* is reduced in complexity and enlarged in size compared to other neomphaline taxa. The lateral swellings of the oesophageal nerve ring, emitting the tentacular nerves, may represent a homologous region to cerebral ganglia in other gastropods; however, their forward placement relative to the origin of the lateral nerve cords confounds positional homology. The cerebral ganglia emit the lateral nerve cords in molluscan tetraneury [49] but in *C. squamiferum* there is a large multi-way junction at the posterior margin of the oesophageal nerve ring that seems to represent a fusion of the typical molluscan ganglia. The medullary nature of the major nerve cords and the enlarged anterior commissure (incorporating the ‘cerebral’ lateral swellings) is reminiscent of the nervous system of Polyplacophora [50].

The single statolith in the pair of statocysts is the same condition as for all other reported instances in Neomphalina [27]. There is a distinct nerve extending to the anterior mantle, positionally equivalent to the nerve extending forward from the osphradial ganglion particularly in *Leptogyra* [27]. The ‘osphradium’ was described as located on the shell muscle in *Rhychopelta* [25], but on the gill stem in *Pachydermia* [26] and in *Chrysomallon*. This level of variability is typical among molluscs and the function (if any) of these structures is entirely speculative [51].

The simultaneous hermaphrodite condition in *C. squamiferum* is so far unique among Peltospiridae [25, 29, 52] and paralleled only among Neomphalina by *Leptogyra* in Melanodrymiidae (known definitively for *L. constricta* Marshall, 1988 and *L. patula* Marshall, 1988; [27]). Early observations suggesting separate males and females in *C. squamiferum* [17] were probably a result of variation in the relative development of the two gonads in different individuals. At the time of the first discovery and report of *C. squamiferum* all known neomphalines (*i.e.* then excluding *Leptogyra*) had separate sexes.

*Chrysomallon squamiferum* also does not have any specialised copulatory organs on the head, which is consistent with other peltospirids [25, 26]. In Melanodrymiidae, *Leptogyra* hermaphrodites have a penis and accessory penis extending from the base of the left cephalic tentacle. All other three melanodrymiid genera have separate sexes and males have copulatory organs [27, 32, 53]. All four genera in family Neomphalidae with available information have separate sexes and the left cephalic tentacle in males is modified into a penis (also *Retiskenea* but the familial placement of this genus is uncertain) [27, 33].

Both gonads in *Leptogyra* share a single gonoduct with the ovary positioned anterior of the testis and the species maybe protandric [32]; by contrast the gonads in *C. squamiferum* are clearly simultaneously present in vertical stacking and two gonoducts appear to fuse into a single genital opening. We have observed the structure previously reported as a ‘spermatophore producing organ’ [17]; however, we note that it is similar in position and structure to the seminal receptical illustrated in *Leptogyra* [27]. Previously, spermatophores in Neomphalina were known from other taxa in Peltospiridae (*Pachydermia laevis*; [17]) and also Melanodrymiidae (*Melanodrymia*; [33]) but not from Neomphalidae.

The selection process leading to the convergent evolution of limpet-form from spirally coiled ancestors has occurred repeatedly among Gastropoda [54, 55]. Shifting from a coiled form to a limpet form has impacts on the positions of organ systems, in particular the relative positions of gonads and digestive gland [56]. In gastropods, the gonad can either be located to the extreme posterior, near the shell apex (the norm for species with a coiled shell) or in an opposite arrangement with the digestive glands at the apex and gonads anterior of it (the norm for limpets) [56]. In *C. squamiferum*, the digestive gland occupies the apex and gonads are displaced anteriorly, and this has previously been attributed to a morphological shift toward the limpet form [17]. Among other Peltospiridae, the anterior gonad is observed in the coiled *Peltospira* and *Chrysomallon*, as well as limpet-formed species (*e.g.*, *Rhynchopelta*, *Echinopelta* [25]); but some, such as *Hirtopelta*, have the opposite arrangement ([25]: fig. 15). Neomphalina as a clade is extremely variable in shell forms ([29]: fig. 7.6). Hence another possible interpretation is that *Chrysomallon* may be secondarily coiled derivation from a recent limpet-form ancestor. Gonad position is, unfortunately, apparently not particularly informative for phylogenetic inference.

Vent invertebrates exhibit a wide range of reproductive traits but tend to have high dispersal ability associated with rapid growth and continuous reproduction [57–59]. Individual hydrothermal vent fields are generally ephemeral and patchy in distribution and vent-endemic invertebrate species must maintain a viable metapopulation across many vent fields to persist and prosper [60, 61]. The patchiness of many deep-sea habitats also means that the chances of larvae successfully colonising a suitable habitat may be low [61, 62]. Displacing the gonads out of the coiled shell apex and into the body whorl provides a larger volume of space for gonads to develop; this may have advantages for *C. squamiferum* in increasing fecundity and in turn increasing the chance of its larvae arriving at a different vent field. Additional inference of gonad index and reproductive quality and fecundity could be determined anatomically [63–66], although we have not attempted this within the present study. *Chrysomallon squamiferum* is presumed to have lecithotrophic larvae with a planktonic dispersal stage like other neomphalines [67], but further aspects of larval dispersal, behaviour, survivability, and metamorphosis are so far largely intractable in deep-sea ecosystems because of the inaccessibility of the living organisms.

### Adaptive significance

This study illustrates how the perception of an organism can be enhanced and fundamentally changed by understanding its internal anatomy. Metazoans in hydrothermal vent environments depend on morphological and physiological adaptations to resolve energetic needs in a food chain based on chemoautotrophy [1, 5, 6, 44]. In this case, we speculate that the derived strategy of housing endosymbiotic microbes in a greatly enlarged oesophageal gland, has been the catalyst for anatomical innovations that serve primarily to improve the fitness of the bacteria, over and above the needs of the metazoan host.

*Chrysomallon squamiferum* lives adjacent to acidic and reducing vent fluid from black smokers or diffuse venting, which contain the chemical and substrates required by the chemoautotrophic bacteria [12, 18, 22]. *Chrysomallon squamiferum* differs distinctly from other deep-sea gastropods, even closely-related neomphalines. The unique external armature of hard dermal sclerites, which are often biomineralised with iron sulfide [19, 20], may help protect the gastropod from the vent fluid, so that its bacteria can live close to the source of electron donors for chemosynthesis. Alternatively, the sclerites may result from deposition of toxic sulfide waste from the endosymbionts, and therefore represent a novel solution for detoxification.

The digestive system of *Chrysomallon* is simple and reduced as nutrition is mainly provided by the endosymbionts, and may be processing solid waste perhaps accidentally ingested. The circulatory system has a huge blood volume and a muscular ventricle that draws blood from the elaborate gill to supply the bacteria. With the significant additional embranchment of the circulatory system in the form of fine blood sinuses within the oesophageal gland, the blood pressure likely decreases to almost zero. The giant ‘dragon heart’ may therefore be necessary to maintain haemocoel circulation throughout the body and particularly the fine blood vessels serving the oesophageal gland where the endosymbionts are housed. This gastropod has no brain, the huge fused neural mass is directly adjacent to and passes through the oesophageal gland where the bacteria are housed. The reproductive system is displaced anteriorly, perhaps enabling greater fecundity. In sum, this dramatic ‘dragon-like’ animal has become a carrying vessel for the survival and propagation of its bacterial endosymbionts.

## Materials and methods

All specimens examined herein were collected from the Longqi vent field [16] (also known as Dragon vent field [13, 14]), Southwest Indian Ridge, 37°47.03'S 49°38.97'E (‘Tiamat Chimney’), depth 2785 m, on-board RRS *James Cook* expedition JC67 using the suction sampler of the remotely operated vehicle (ROV) *Kiel 6000* [13]. *Chrysomallon squamiferum* was densely populated in the areas immediately surrounding diffuse-flow venting, seen visually as shimmering water. The specimens were fixed and stored in 4 % buffered formalin upon retrieval on-board the ship.

10 adult specimens were dissected with the aid of stereo microscopes (Olympus SZX9, SZX16) and photographs were taken using a digital single lens reflex (DSLR) camera (Olympus E-600) mounted to the microscope trinocular.

One specimen with shell and mantle removed was freeze-dried overnight and Scanning Electron Microscopy (SEM) was undertaken using a Hitachi TM3000 table-top SEM (British Antarctic Survey, Cambridge), to capture the structural details of head and ctenidium. As the specimen was large 22 micrographs were stacked in the software Adobe Photoshop CS4 to compose the final image.

One of the smallest *C. squamiferum* juvenile specimens ever collected (shell length ca. 3 mm) was selected for serial sectioning and 3D tomographic reconstruction. The selected juvenile specimen was decalcified in 2 % EDTA (pH 7.2) for 48 hours, followed by subsequent acetone dehydration series, embedding, and tomographic model reconstruction using the software AMIRA v.5.3.3 (FEI Visualisation Sciences Group) as described by Ruthensteiner [68].

Prior to embedding, the specimen was stored in diluted Epon epoxy resin mixture (1:1 with 100 % acetone) overnight at room temperature unlidded, allowing acetone to evaporate. The specimen was then embedded in Epon with DPM-30 accelerator for a further 24 hours at 60 °C, according to the manufacturer’s instructions (Sigma). Samples were serially sectioned at a thickness of 1.5 μm using an automated rotary microtome (Leica RM2255) fitted with a diamond knife (HistoJumbo 8 mm, DiATOME, Switzerland). Sections were stained using the high contrast monochromatic methylene blue-azure II stain [69] and cover-slipped using Araldite resin following manufacturer’s instructions (Agar Scientific).

The serial semithin sections of the complete animal included 1700 sections; a subsample of every third section throughout the entire specimen was digitally captured using a DSLR camera (Olympus E-600) mounted to a compound microscope trinocular (Olympus BX41), at an appropriate magnification to maximise specimen visibility. The resulting images were processed in Adobe Photoshop CS4 for contrast enhancement, size reduction, and converted to greyscale. The processed images were imported into Amira v5.3.3 and aligned into a single stack. Materials of interest were highlighted digitally throughout the stack for 3D visualisation and the final tomographic model was produced by post-processing including surface rendering and smoothing. The resulting semithin sections are deposited in Zoologische Staatssammlung München (Munich, Germany), with catalogue number ZSM 20151000.

## Additional file

Additional file 1: Figure S1. 3D tomographic reconstruction of the ‘scaly-foot gatropod’, *Chrysomallon squamiferum*, as a full interactive model. The model can be accessed by clicking into the figure (Adobe Acrobat Reader v7 or higher). Left click and drag to rotate, hold down the ‘control’ key while doing so to move, and hold down ‘shift’ to zoom. The dropdown menu in the floating window or the view pane in the model tree can be used to switch between pre-saved views. Components can also be activated or de-activated by toggling the checkbox in the model tree. The 3D PDF was generated using Adobe Acrobat Pro XI by importing .u3d files, converted from .obj exports of AMIRA v5.3.3 surface files.
