# Supplementary material for: The heart of a dragon: 3D anatomical reconstruction of the ‘scaly-foot gastropod’ (Mollusca: Gastropoda: Neomphalina) reveals its extraordinary circulatory system
Source: Front Zool. 2015 Jun 18;12:13. doi: 10.1186/s12983-015-0105-1 (PMC4470333; doi:10.1186/s12983-015-0105-1)
Supplement: Additional file 1: Figure S1. — 3D tomographic reconstruction of the ‘scaly-foot gatropod’, Chrysomallon squamiferum, as a full interactive model. The model can be accessed by clicking into the figure (Adobe Acrobat Reader v7 or higher). Left click and drag to rotate, hold down the ‘control’ key while doing so to move, and hold down ‘shift’ to zoom. The dropdown menu in the floating window or the view pane in the model tree can be used to switch between pre-saved views. Components can also be activated or de-activated by toggling the checkbox in the model tree. The 3D PDF was generated using Adobe Acrobat Pro XI by importing .u3d files, converted from .obj exports of AMIRA v5.3.3 surface files. [file 12983_2015_105_MOESM1_ESM.pdf]

Additional file 1: Figure S1.

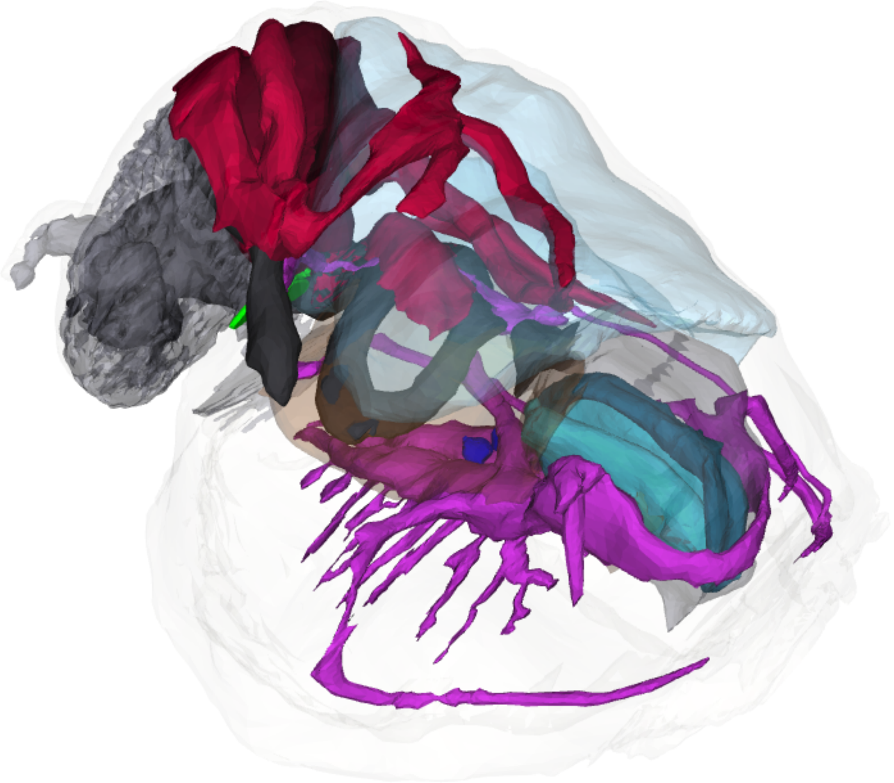

3D tomographic reconstruction of the ‘scaly-foot gatropod’, full interactive model. The interactive model can be accessed by clicking into the figure (Adobe Acrobat Reader v7 or higher). Hold left click and drag to rotate, hold down ctrol while doing so to move, and hold down shift while doing do to zoom (alternatiavely hold right click and drag or use mouse wheel). Switch between pre-saved views using the dropdown menu in the floating window or click on the view pane in the model tree. Components can also be activated or disactivated by toggling the checkbox in the model tree. The 3D PDF was generated using Adobe Acrobat Pro XI by importing .u3d files, converted from .obj exports of Amira v5.3.3 surface files.
